# Quality of Life of Saudi Women With Chronic Lower Urinary Tract Symptoms

**DOI:** 10.7759/cureus.32439

**Published:** 2022-12-12

**Authors:** Mohammed AlAteeq, Saeed AlSary, Joud AlBaraki, Manar AlMutairi, Noura AlEnazi, Shadin AlDhalaan, Someiah AlYahya, Nazish Masud

**Affiliations:** 1 Family Medicine Department, Ministry of National Guard-Health Affairs, King Abdullah International Medical Research Center, King Saud Bin Abdulaziz University for Health Sciences, Riyadh, SAU; 2 Obstetrics and Gynecology Department, Ministry of National Guard-Health Affairs, King Abdullah International Medical Research Center, King Saud Bin Abdulaziz University for Health Sciences, Riyadh, SAU; 3 Family Medicine, King Saud Bin Abdulaziz University for Health Sciences, Riyadh, SAU; 4 Department of Biostatistics, Epidemiology and Environmental Health Sciences (BEES), Jiann-Ping Hsu College of Public Health, Georgia Southern University, Georgia, USA

**Keywords:** ipss, general health, urinary incontinence, urogynecology, women health

## Abstract

Background

Chronic lower urinary symptoms (LUTS) are reported to affect more than half of women of all ages and have a significant impact on their quality of life (QoL). We aimed in this study to assess the QoL of adult Saudi women with chronic LUTS.

Methods

A cross-sectional study was done on 390 female patients diagnosed with LUTS at three tertiary care hospitals in Riyadh, Saudi Arabia, from October to December 2021. LUTS are classified into three categories: symptoms related to bladder storage (increased daytime frequency, nocturia, and urinary incontinence [UI]), symptoms of bladder voiding (hesitancy, extended micturition time, and insufficient emptying), and symptoms involved in the post-urination phase such as post-micturition dribbling. The data was collected using a self-administered questionnaire which comprised demographic characteristics, International Prostate Symptom Score (IPSS) for assessment of LUTS severity, and King’s Health Questionnaire (KHQ) for assessment of QoL.

Results

After analyzing the study results, we found that symptoms were mild, moderate, and severe in 11%, 51%, and 39% of participants, respectively. Increased age and parity were found to have a significant association with increased symptom severity (*p*-value <0.05).

The current study reported a moderate effect of LUTS on QoL. There was a significant correlation between increased symptom severity and negative impact on QoL, excluding the personal relationships domain. The highest scores in KHQ, which indicate worse QoL, were found in the domains of incontinence impact and emotions, while the lowest scores, which indicate better QoL, were found in the domains of social limitations and severity measures.

Employed females were found to have worse QoL in the severity measures domain, which refers to the degree of urinary symptoms affecting day-to-day functioning. In addition, younger age, below 45 years, was found associated significantly with better QoL, especially in the domains of general health, personal relationships, and severity measures.

Conclusion

In the current study, the majority of patients reported moderate symptoms severity. Chronic LUTS have a significant impact on the QoL of Saudi women in many aspects, including physical, social, and emotional health, especially for those who have moderate to severe symptoms. Healthcare providers should assess high-risk women for the presence of LUTS. Furthermore, we recommend evaluating the QoL of patients with LUTS as a part of routine management.

## Introduction

Lower urinary tract symptoms (LUTS) have been defined as qualitative pathological changes of the lower urinary tract that are subjectively noticed by an individual, their caretaker, or their partner, leading them to seek medical attention [1]. LUTS are classified into three categories: symptoms related to bladder storage (increased daytime frequency, nocturia, and urinary incontinence [UI]), symptoms of bladder voiding (hesitancy, extended micturition time, and insufficient emptying), and symptoms involved in the post-urination phase such as post-micturition dribbling [1]. Other defined symptoms are related to sexual intercourse, prolapsing of pelvic organs, and pain in the genitals and lower urinary tract area [1].

Many factors have been identified as risk factors for LUTS. These include age, pregnancy, parity, menopause, uterine prolapse, hysterectomy, obesity, diet, fluid and caffeine intake, chronic coughing, constipation, and family history [2-5].

LUTS are very prevalent and tend to occur more among women compared to men. In 2008, an estimation of LUTS prevalence examined gender- and age-stratified prevalence data from the European Perspective Investigation into Cancer and Nutrition (EPIC) study along with gender- and age-stratified worldwide and regional population estimates from the US Census Bureau International Data Base. The results showed that 1.9 billion LUTS cases were reported internationally [6].

Locally, a study was done in the Al-Qassim region, Saudi Arabia, on 180 young individuals and found that the prevalence of LUTS is high among young adults and particularly in women, with 52.7% being females [7]. Similar findings were reported in a study investigating the prevalence of LUTS among patients diagnosed with multiple sclerosis. The study found that more women reported LUTS than men [8].

LUTS have been found in several studies to cause a remarkable impact on the quality of life (QoL) of both genders, with negative effects on many aspects like physical, psychological, social, and sexual life [9]. A recent study reported that individuals with LUTS have a lower QoL and are more likely to be depressed or anxious [10]. It also results in a financial burden due to the cost of additional incontinence supplies and laundry, as well as an influence on sexual function, both of which appear to be potential sources of reduced QoL [11].

According to Al-Badr et al., 29.9% of Saudi women with UI reported limitations in social activities, and 17.8% had adverse effects on their sexual relationships [12]. A cross-sectional study in the US, UK, France, Spain, and Germany found that patients with overactive bladder (OAB) and UI had lower general and disease-specific QoL, a lower likelihood of having a job, and decreased productivity [13]. A study conducted by Kogan et al. in Russia, the Czech Republic, and Turkey showed that LUTS were found in 84% of women and resulted in mild effects on their QoL [14]. Robertson et al. reported that the QoL of patients with moderate LUTS was equivalent to that of patients with chronic conditions such as hypertension, diabetes, or cancer, while patients with severe LUTS had a similar QoL to those with a heart attack or stroke [5]. Despite the high prevalence of LUTS among women, there is insufficient data locally and regionally to answer the question: What is the QoL of Saudi women who have chronic LUTS? Therefore, this study aimed to assess the QoL of adult Saudi women with chronic LUTS.

## Materials and methods

Study settings and participants

This cross-sectional study was conducted on patients followed up in urogynecology clinics at three tertiary hospitals in Riyadh, Saudi Arabia: King Abdulaziz Medical City (KAMC), King Fahad Medical City (KFMC), and Prince Sultan Military Medical City (PSMMC), from October to December 2021. Eligible patients were female patients aged 18 years and above diagnosed with chronic LUTS. Pregnant women, women who had undergone pelvic surgery or radiation, or women who were diagnosed with congenital anomalies were excluded.

The sample size was calculated based on an 88% prevalence of LUTS reported in a regional study by Mourad et al. [15]. The reduction in QoL was assumed to be 50%. Using the 95% confidence interval and 5% margin of error, the calculated sample size was estimated to be 385 participants, which was adjusted to 400 participants to compensate for incomplete questionnaires. The sample size was calculated using the Raosoft sample size calculator (Seattle, Washington), which can be accessed online freely.

Data collection

Using a non-probability convenience sampling technique, patients were approached at the clinics’ waiting areas and given the questionnaires with a written consent form. The questionnaire was composed of three parts. The first part was for demographic data (age, marital status, parity, educational level, job, income, history of chronic illness, and physical activity), the second part was for assessment of the severity of LUTS, and the third part was for assessment of QoL. To assess the severity of LUTS, we used the Arabic version of the International Prostate Symptom Score (IPSS) [16]. Although IPSS was developed and used to assess LUTS in men with prostate hyperplasia, it can be used for women with LUTS due to other causes. Several studies reported the validity of IPSS use in women [17-19]. The IPSS inventory contains seven questions to measure the severity of LUTS, with a scale of 0 to 5 for each question. Severity was categorized according to the sum of the seven questions’ scores as mild (score 0-7), moderate (8-19), and severe (20-35).

For the assessment of QoL, a translated version of King’s Health Questionnaire (KHQ) was used [20]. KHQ contains 21 items investigating nine domains. The first domain has two questions about general health perception and incontinence impact. The second part contains 18 questions in eight domains: role limitations, physical limitations, social limitations, personal relationships, emotions, sleep/energy, and severity measures. The scoring of the domains ranges from 0 (best) to 100 (worst). The questionnaire was translated from English to Arabic and then back-translated to English by an independent agent to ensure accuracy. The translated questionnaire was reviewed by two experts, one in the specialty of family medicine and one in biostatistics. A pilot study was done with 20 women and resulted in the rephrasing of the “personal relationships” domain.

Statistical analysis

For data entry and analysis, IBM Corp. Released 2013. IBM SPSS Statistics for Windows, Version 22.0. Armonk, NY: IBM Corp. was used. A descriptive analysis of normally distributed numerical variables was done, and the results are reported in terms of the mean and standard deviation. Skewed numerical variables are reported as the median, first quartile, and third quartile. Categorical variables such as marital status and educational level were described using frequencies and percentages.

The relationship between the severity of LUTS and IPSS scores was analyzed using the chi-squared test. The correlation between the severity of LUTS and KHQ scores of all domains was analyzed by the Kruskal-Wallis test. The association between KHQ scores and patients' demographic profiles was evaluated by the Mann-Whitney U test. We considered p-values less than 0.05 to be statistically significant. 

Ethical approval

Ethical approval was obtained from King Abdullah International Medical Research Center (KAIMRC), study number SP21R/172/04, dated May 23, 2021. Patients who voluntarily participated were guaranteed privacy, confidentiality, and anonymity and that their participation would not affect their medical care. Upon administering the questionnaire, written consent was obtained from each participant. The investigators maintained the questionnaires and assigned them three-digit serial numbers. Participants could not be traced after collecting the data. Information obtained from data sheets was used for research purposes only. The study was carried out according to the principles of the Declaration of Helsinki.

## Results

Patient demographics

Out of 400 patients enrolled, 10 patients were excluded due to incomplete data, and the analysis was done with the remaining 390 patients. The mean age was 51 years (SD, 12 years), 290 (74%) patients were currently married, and 95% of married patients had children with a mean of six children (SD, three children). Approximately one-third of the study subjects had diabetes, hypertension, and/or joint diseases. Table 1 summarizes the demographics of the participants.

**Table 1:** Profile of patients (n=390) Only "Yes" percentages are presented in the table for simplicity

Variable	Mean (±SD)	
Age (years)	51 (±12)	
Parity	6 (±3)	
	Category	N (%)
Marital status	Married	290 (74)
Children	Yes	370 (95)
Level of education	No education	114 (29)
	Primary	71 (18)
	Intermediate	28 (7)
	High school	74 (19)
	Bachelor	92 (24)
	Higher degree	11 (3)
Income	<3000	85 (26)
	3000-6000	84 (25)
	6000-9000	35 (11)
	9000-12000	54 (16)
	12000-15000	38 (12)
	>15000	35 (11)
Employment	Yes	80 (21)
Job	Teacher	27 (37)
	Administration	12 (16)
	Healthcare worker	8 (11)
	Other	26 (36)
Morbidities	Diabetes	117 (30)
	Hypertension	116 (30)
	Joint Disease	112 (29)
	Cardiovascular disease	52 (13)
	Asthma	37 (10)
	Dyslipidemia	29 (7)
	Hypothyroidism	28 (7)
	Previous or current tumor	14 (4)
	Mental disorder	11 (3)
	other	29 (7)

Descriptive summary of symptom severity and QoL

**Figure 1:**
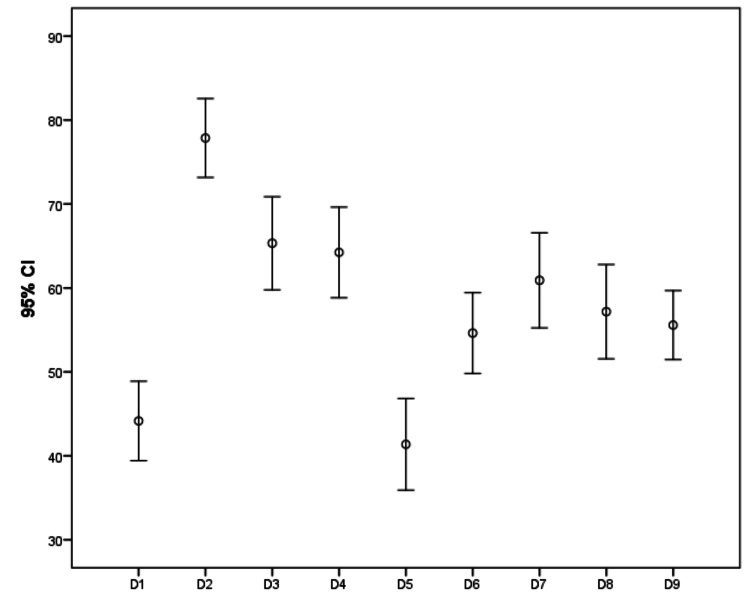
Patient's quality of life scoring according to King’s Health Questionnaire D1: General health perception, D2: Incontinence impact, D3: Role limitations, D4: Physical limitations, D5: Social limitations, D6: Personal relationships, D7: Emotions, D8: Sleep/Energy, D9: Severity measures

**Figure 2:**
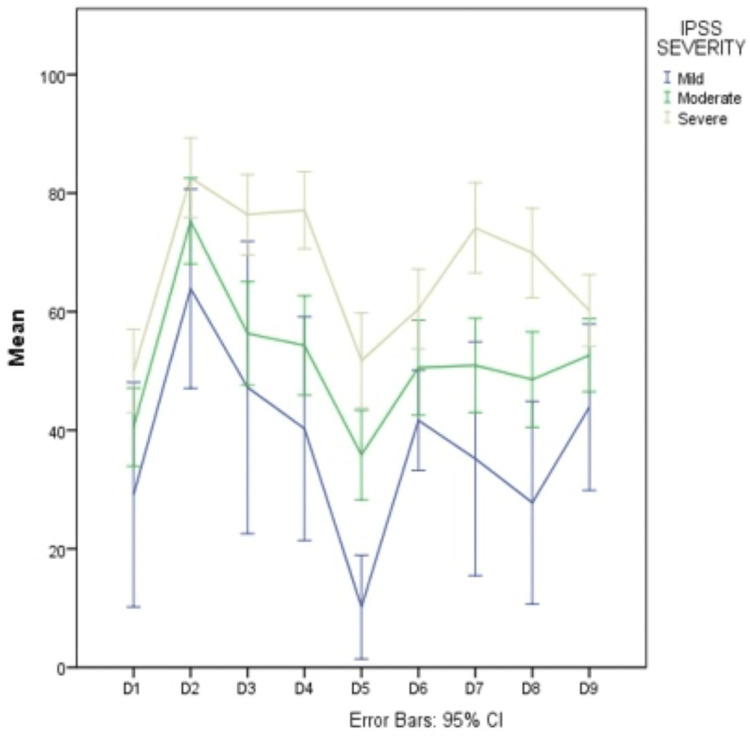
QoL domains scoring according to KHQ in the three groups of the IPSS severity scale KHQ: King’s health questionnaire, IPSS: International prostate symptoms score

**Figure 3:**
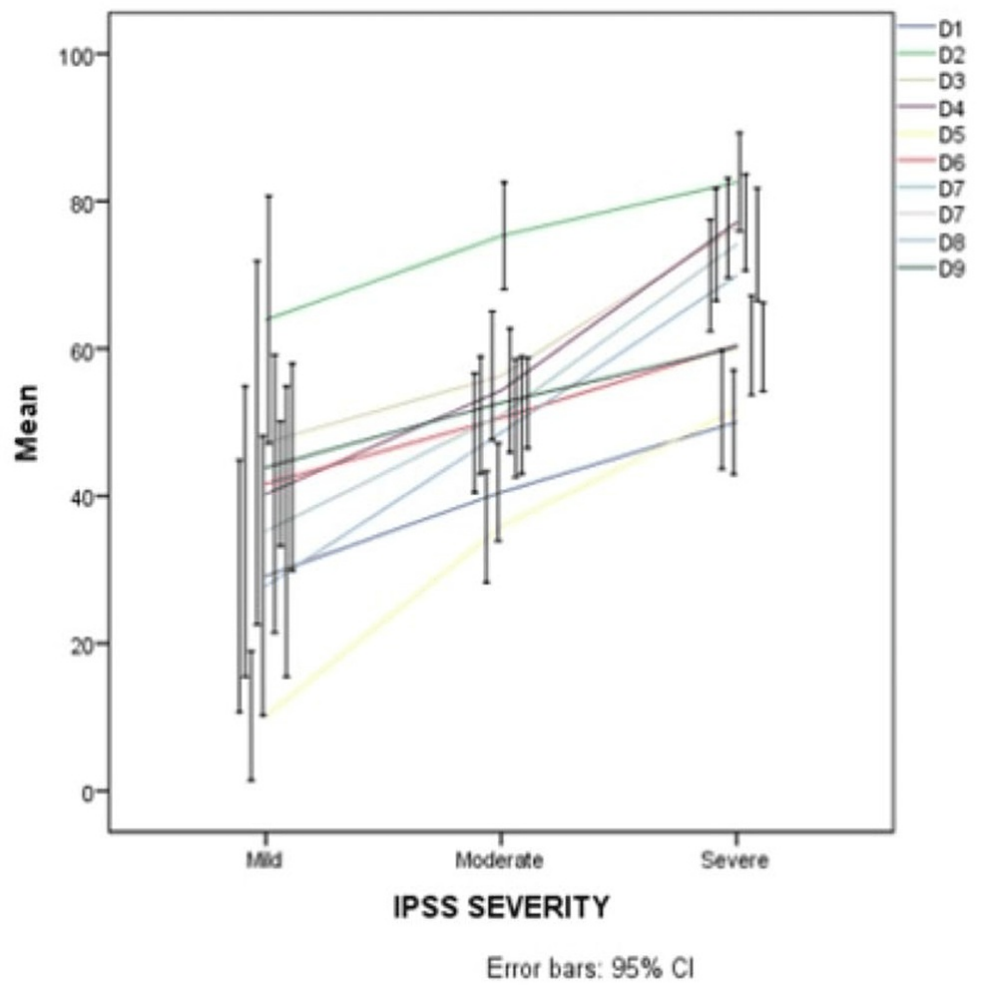
QoL domains scoring at KHQ in the three groups of the IPSS severity scale IPSS: International prostate symptom score

In regard to severity, according to IPSS, symptoms were mild, moderate, and severe in 11%, 51%, and 39% of participants, respectively. For all participants, the highest scores in KHQ, which indicate worse QoL, were found in the domains of incontinence impact and emotions, while the lowest scores, which indicate better QoL, were found in the domains of social limitations and severity measures. All groups of IPSS severity demonstrated a significant correlation with QoL domain scoring with a p-value of 0.001, i.e., more severe symptoms imply worse QoL, except in the domain of personal relationships (P-value = 0.039) (Table 2 and Figures 1-3).

**Table 2:** QoL domains scoring at KHQ in all participants and in the three groups of IPSS severity scale (n=390) Ϯ (N=137) Those with zero scores were treated as missing variable IPSS: International prostate symptom score

Domains	All patients	IPSS SEVERITY			P-value
		Mild(n=41)	Moderate(n=198)	Severe(n=151)	
	Median (Q1-Q3)	Median (Q1-Q3)	Median (Q1-Q3)	Median (Q1-Q3)	
D1: General health perception	50 (25-50)	25 (0-50)	50 (25-50)	50 (25-75)	<0.001
D2: Incontinence impact	67 (67-100)	33 (33-67)	67 (33-100)	100 (67-100)	<0.001
D3: Role limitations	50 (33-100)	17 (0-50)	42 (17-67)	83 (50-100)	<0.001
D4: Physical limitations	50 (17-83)	17 (0-33)	50 (17-67)	83 (50-100)	<0.001
D5: Social limitations	33 (11-67)	0 (0-22)	22 (0-56)	67 (33-89)	<0.001
D6: Personal relationships Ϯ	50 (33-83)	33 (33-50)	42 (17-83)	67 (33-83)	0.039
D7: Emotions	56 (22-100)	22 (0-56)	44 (11-67)	89 (56-100)	<0.001
D8: Sleep/energy	50 (17-83)	17 (0-33)	33 (17-67)	83 (50-100)	<0.001
D9: Severity measures	47 (27-67)	27 (13-47)	40 (20-60)	60 (40-80)	<0.002

Symptoms severity and patient demographics

In regard to the association of symptom severity with patient demographics, age, parity, and exercise level were found to have statistically significant associations with symptom severity (p-value <0.05) (Table 3).

**Table 3:** Association of IPSS severity with the demographic profile of patients All the percentages have been rounded to the nearest 10. *Chi square, fisher's exact and Kruskal–Wallis test applied as applicable. Significant p-values presented as bold. IPSS: International prostate symptoms score

		IPSS SEVERITY			
		Mild(n=41)	Moderate(n=198)	Severe(n=151)	p-value*
		Median(Q1-Q3)	Median(Q1-Q3)	Median(Q1-Q3)	
Age		45(39-51)	51(43-57)	54(44-60)	<0.001
Parity		5(3-8)	6(4-8)	6(5-9)	0.01
		N (%)	N (%)	N (%)	
Children	No	4 (20)	8 (40)	8 (40)	0.32
	Yes	37 (10)	190 (51)	143 (39)	
Level of education	No education	6 (5)	58 (51)	50 (44)	0.28
	Primary	6 (9)	33 (47)	32 (45)	
	Middle	4 (14)	16 (57)	8 (29)	
	High school	11 (15)	34 (46)	29 (39)	
	Bachelor	13 (14)	52 (57)	27 (29)	
	Higher degree	1 (9)	5 (46)	5 (46)	
Income	<3000	10 (12)	38 (45)	37 (44)	0.65
	3000-6000	4 (5)	44 (52)	36 (43)	
	6000-9000	4 (11)	20 (57)	11 (31)	
	9000-12000	7 (13)	30 (56)	17 (32)	
	12000-15000	5 (13)	21 (55)	12 (32)	
	>15000	5 (14)	17 (49)	13 (37)	
Employment	No	32 (10)	151 (49)	127 (41)	0.19
	Yes	9 (11)	47 (59)	30 (30)	
Job	Teacher	2 (7)	16 (59)	9 (33)	0.55
	Admin	1 (8)	6 (50)	5 (42)	
	Healthcare worker	0 (0)	5 (63)	3 (38)	
	Other	5 (19)	16 (62)	5 (19)	
Exercise	Do not exercise	4 (4)	41 (45)	47 (51)	0.04
	Light	16 (11)	77 (51)	59 (39)	
	Moderate	17 (15)	63 (55)	34 (30)	
	Vigorous	4 (13)	17 (53)	11 (34)	

QoL and patient demographics

There was a significant difference in the domains of health perception, personal relationships, and severity measures between the age groups below 45 and above 45 years. There was also a significant difference in social limitations and sleep/energy domains between married and unmarried women. Finally, in only the severity measurements domain, there was a significant difference between employed and unemployed women (Table 4).

**Table 4:** Association of QoL (at KHQ) with some demographic profiles of patients * Mann-Whitney U test applied, bold represents the significant p-values KHQ: King’s health questionnaire

		D1	D2	D3	D4	D5	D6	D7	D8	D9
		Median (Q1-Q3)	Median (Q1-Q3)	Median (Q1-Q3)	Median (Q1-Q3)	Median (Q1-Q3)	Median (Q1-Q3)	Median (Q1-Q3)	Median (Q1-Q3)	Median (Q1-Q3)
Age (years)	45 and below	50 (0-50)	67 (67-100)	50 (33-100)	67 (33-83)	22 (0-67)	67 (33-83)	56 (22-78)	33 (17-67)	53 (33-73)
	46 and above	50 (25-75)	67 (67-100)	50 (17-100)	50 (17-83	33 (11-67)	33 (33-67)	56 (22-100)	50 (17-83)	40 (20-60)
	p-value*	0.03	0.58	0.71	0.13	0.07	0.02	0.30	0.28	0.01
Marital status	Unmarried	50 (25-75)	67 (50-100)	58 (17-100)	58 (33-83)	33 (22-78)	50 (33-67)	67 (22-100)	67 (33-100)	47 (27-67)
	Married	50 (25-50)	67 (67-100)	50 (33-100)	50 (17-83)	33 (0-67)	50 (33-83)	56 (22-89)	33 (17-83)	47 (27-67)
	p-value*	0.27	0.67	0.70	0.45	0.01	0.91	0.11	0.01	0.96
Children	No	25 (0-63)	67 (33-100)	50 (8-100)	58 (17-92)	33 (0-72)	33 (33-42)	56 (28-100)	58 (25-100)	47 (37-60)
	Yes	50 (25-100)	67 (67-100)	50 (33-100)	50 (33-83)	33 (11-67)	50 (33-83)	56 (22-100)	50 (17-83)	47 (27-67)
	p-value*	0.39	0.46	0.94	0.98	0.88	0.27	0.65	0.45	0.82
Employment	No	50 (25-75)	67 (67-100)	50 (33-100)	50 (17-83)	33 (11-67)	50 (33-83)	56 (22-100)	50 (17-83)	43 (27-67)
	Yes	50 (25-50)	67 (33-100)	50 (33-83)	58 (33-83)	33 (0-56)	33 (33-67)	44 (22-78)	50 (25-67)	53 (33-73)
	p-value*	0.39	0.15	0.68	0.48	0.14	0.30	0.21	0.60	0.02

## Discussion

Although LUTS is prevalent among women, there are limited data about its severity and effect on QoL among Saudi women. The current study provides new findings in this area. In regard to the severity of LUTS, the majority of patients in this study (51%) reported moderate symptoms. For comparison, moderate to severe symptoms were reported by 23.9% of Brazilian women [15]. In a survey done on Chinese individuals, much lower rates of severity were reported [21]. This is probably related to different methodologies in that the previous study was done on a general population, while the current study was done on visitors to a specialized clinic.

In the current study, the severity of LUTS was significantly correlated with parity. Those who reported severe symptoms were found to have a median of six (5-9) children. Similarly, in a study conducted with Danish women, parity was found to be associated with an increased risk of LUTS, except for urge incontinence [22]. An Egyptian study reported the same correlation [23].

Age was also found to correlate positively with the severity of LUTS in the present study. Consistent findings were reported by a Korean study, which revealed deteriorated voiding function in women with stress UI at an advanced age. In addition, it showed a decrease in the detrusor contractility index and an increase in post-voiding residual urine volume, which indicates worsening of symptoms, especially in women older than 60 years [24]. Another study on patients aged 70 years and older reported that patients with severe UI were more likely to be older women with self-perceived health status, significant disability, and more depressive symptoms [25].

A cross-sectional study about the risk factors of LUTS in adult Chinese women showed that numerous factors, such as advanced age and coexisting pelvic organ prolapse, were significant predictors of such symptoms [26]. This clarifies the deterioration of bladder and urethral function as a result of the aging process, which explains why the severity of LUTS increases with aging. Several studies have reported that some voiding symptoms are significantly related to advanced age, including straining to void, hesitancy, and intermittency [23-24, 26-31]. 

The most frequent chronic medical conditions reported by participants in the current study were diabetes mellitus, hypertension, and joint disease. The reported rates are similar to the reported prevalence in the general population in Saudi Arabia [31,32]. Many comorbidities and risk factors are associated with the risk of chronic LUTS [4]. A study was done in Ontario, Canada, to determine the prevalence and correlates of UI in older adults living with T2DM receiving home care services. The study found that diabetes mellitus was a substantial risk factor [33]. Other studies reported similar findings [34].

In regard to hypertension, to our knowledge, no study has indicated hypertension as a risk factor for LUTS. This may be explained by the side effects of frequently used hypertension medications. Diuretics can cause polyuria, frequency, and urgency, while alpha-blockers can cause urethral relaxation. The mechanism by which hypertension is associated with LUTS should be evaluated further. The association of arthritis with LUTS indicates the possibility of an inflammatory process. Abushamma et al. reported that 94.4% of patients with rheumatoid arthritis had at least one symptom of LUTS [35]. However, the reason behind it is yet to be explained.

The present study shows that the level of physical activity is inversely associated with the severity of LUTS. A prospective study looking into the association between exercise and LUTS in parous women found that women who exercise vigorously had the lowest possibility of developing stress incontinence after three years of follow-up. After 11.5 years of follow-up, it was found that women with the highest level of physical activity have the lowest rates of developing stress, urgency, and mixed incontinence compared to women who do not exercise [36]. A controlled clinical trial conducted with women who have multiple sclerosis and LUTS found that after six months of physical activity, participants showed improvements in LUTS and QoL [37]. On the other hand, a study measuring the impact of exercise on stress incontinence found that there was a significant positive correlation between stress UI and intense exercise [38].

The current study reported a moderate effect of LUTS on QoL. The most affected domains were incontinence impact and emotions. Incontinence impact was evaluated by asking participants about the life burden due to disease, and the feedback demonstrated the heavy burden of LUTS on a patient’s life. Participants were asked about depression, anxiety, and feeling bad, and the results suggest that screening is required for depression and anxiety in this group of patients.

A similar study was done in Spain using KHQ to assess the QoL of women with UI and a mean age of 57 years. The study found the best scores on the scale of personal relationships with a mean score of 26.8, whereas the worst scores were noted in the domain of impact of UI, which had a mean score of 82.96 [39]. Another study done with older women aged 70 years and above with UI found impaired QoL, and the effect increased with increases in the severity of symptoms [25]. Similarly, a significant reduction of health-related QoL was reported among patients with UI in a multinational cross-sectional survey in the US, the UK, France, Spain, and Germany [13]. Several other studies reported the same findings [9-11,21,23,40].

The Epidemiology and Impact of Urinary Incontinence (EPIC) survey in Russia, the Czech Republic, and Turkey found that LUTS were associated with relatively modest effects on QoL and work performance in the majority of individuals [14]. A local study was done to investigate QoL among older patients with UI using a different tool from the KHQ used in the current study. The study found limitations of social life in 36.3% of patients, a negative impact on physical activity in 18.5%, and a negative impact on personal hygiene in 21.8% [34]. A Chinese study investigated QoL among men and women with UI and found a significant decrease in transformed physical health scores from 74.8 for those with no nocturia to 49.5 [41].

Chronic urinary symptoms not only affect QoL but may also put patients at high risk of depression. Nygaard reported that women avoid social and physical activities due to UI, which leads to social isolation and depression. Nygaard found also that women with severe and mild-moderate incontinence were 80% and 40% more likely to have depression than continent women, respectively [42]. A similar finding was reported in a Chinese study [21].

Younger age in the current study was found to be significantly associated with better QoL, especially in the domains of general health, personal relationships, and severity measures. Similar findings were reported in another study [39]. This finding can be explained by the severity of LUTS, as younger women were found to have less severe symptoms.

In this study, employed females were found to have worse QoL in the severity measures domain, which refers to the degree of urinary symptoms affecting day-to-day functioning. Similarly, a study conducted with 8284 patients across China, Taiwan, and South Korea reported a significant impact on work ability in patients with stress UI, nocturia, and overactive bladder across measures in a work limitation questionnaire (starting work, continuing work, concentrating on work, interacting with people, and completing work) [41].

One aspect of QoL that is particular to Muslim women and men is daily prayers. Ablution after urination is needed before performing prayers, and in the case of UI, it must be done before each prayer (i.e., five times a day). This may result in distress and impairment of QoL. In a local study by AlBader, 14.5% of participants reported an issue with prayer, and 33.8% reported an inability to pray on time [12]. This issue can negatively affect the QoL of Muslim women as prayers are an essential daily activity for them. A similar effect was reported for Qatari women: 64% of them had difficulty performing daily prayers, while the number increased to 90% in the UAE and Egyptian studies [23,40,43]. Despite the negative effect of LUTS on QoL, there were high rates of women not seeking medical treatment or health advice. This was attributed to different reasons in several studies, like feeling embarrassed, thinking about it is a normal change with age, and not believing in medication benefits [10,12,15,40].

Limitations

One of the limitations of this study is that it is a cross-sectional study. Furthermore, some data were based on self-reports like medical conditions and level of physical activity. The study did not investigate the level of control of comorbidities that may affect symptoms severely, like diabetes mellitus and hypertension.

## Conclusions

LUTS have a significant impact on the QoL of Saudi women in many aspects, including physical, social, and emotional health, especially for those who have moderate to severe symptoms. Age, parity, and physical activity were found to have significant effects on the severity of LUTS. Based on these findings, primary healthcare providers should assess high-risk women for the presence of LUTS. Furthermore, we recommend evaluating the QoL of patients with LUTS as a part of routine management.
